# Research Advances of Modification and Nutraceutical Properties of Polysaccharide

**DOI:** 10.3390/foods12163065

**Published:** 2023-08-15

**Authors:** Shuang Song, Chunqing Ai

**Affiliations:** National Engineering Research Center of Seafood, School of Food Science and Technology, Liaoning Key Laboratory of Food Nutrition and Health, Dalian Polytechnic University, Dalian 116034, China; acqdongying@163.com

As a group of important biopolymers, polysaccharides exist widely in living organisms and play many known and unknown biological roles in life activities via different pathways. In recent years, the gut microbiota has been considered an important organ and could play a critical role in host health and diseases, and emerging evidence indicates that dietary polysaccharides are effective for the modulation of gut microbiota [1]. In addition, the modification of polysaccharides could alter or enhance their nutraceutical properties, which may extend their applications. Notably, the nutraceutical properties of polysaccharides are dependent on their chemical structures and chain conformations [2], and thus the structural identification of polysaccharides and their derivatives is helpful for their development and application in the food and pharmaceutical fields. In this Special Issue, different modification (e.g., molecular weight reduction and chemical and metal ion modification) are reported as the efficient upregulation of polysaccharide nutraceutical properties.

Molecular weight considerably affects the potent biological activities of polysaccharides, and low-molecular-weight polysaccharides often have better biological activity. Liu et al. [3] explored the different molecular weights of *Gracilaria lemaneiformis* polysaccharides (GLPs) on antioxidant activities and cytotoxicity. GLPs, especially GLP-7 with 2.42 kDa, had good antioxidant activities and protective effects against calcium-oxalate-induced HK-2 cell damage, which may be potential stone preventive drugs. Qi et al. [4] illustrated that the photocatalytic method could be applied to obtain low-molecular-weight fucoidans, DF-0.5 (average MW 90 kDa) and DF-3 (average MW 3 kDa), which did not strip the sulfate groups. In addition, DF-3 could only prolong the clotting time of APTT, suggesting its selective participation in the intrinsic pathway of the blood coagulation cascade. Xu et al. [5] investigated that partially degraded konjac glucomannan could reduce the food intake of rats by enhancing satiety to inhibit excessive weight gain.

To improve the biological activities of polysaccharides, some mental ions, such as selenium and iron, could be used for preparing sugar complex formation. For example, Zhang et al. [6] reported that a novel pumpkin peel polysaccharide–chromium (III) complex (PPP-Cr (III)) with a Cr content of 23.77 mg/g was synthesized successfully, and the binding sites were O-H and C=O. Moreover, compared with the original polysaccharide, PPP-Cr (III) showed better effects on the regulation of the AMPK/GSK-3β signaling pathway to induce better hypoglycemic activity. Li et al. [7] fabricated the selenylation of *Portulaca Oleracea* L. polysaccharide, which could enhance its anti-cancer effect. In addition, Jiang et al. [8] prepared *p*-hydroxybenzoic-acid-grafted chitosan film which demonstrated comprehensive performance improvements and has potential as a novel packaging material for the preservation of fresh-cut fruits and vegetables.

Two review articles were also collected in this Special Issue. Cao et al. [1] summarized the modulation of *Lycium barbarum* polysaccharides on gut microbes and related metabolites. Jiao et al. [2] also discussed the effects of modification on the bioactivities of pectin.

Overall, these articles widen the information on the modification and nutraceutical properties of polysaccharides, promoting the development of polysaccharides in the food industry. Finally, we would like to thank all these authors for their submissions and the referees for their valuable comments.
